# Prognostic impact of the glypican family of heparan sulfate proteoglycans on the survival of breast cancer patients

**DOI:** 10.1007/s00432-021-03597-4

**Published:** 2021-03-19

**Authors:** Paulina Karin Grillo, Balázs Győrffy, Martin Götte

**Affiliations:** 1 Department of Gynecology and Obstetrics, Münster University Hospital, Albert-Schweitzer-Campus 1, 11, 48149 Münster, Germany; 2 Department of Bioinformatics, Semmelweis University, Budapest, Hungary; 3 2nd Department of Pediatrics, Semmelweis University, Budapest, Hungary; 4 TTK Momentum Cancer Biomarker Research Group, Budapest, Hungary

**Keywords:** Breast cancer, Prognosis, Glypicans, Proteoglycans, Gene expression, Survival analysis

## Abstract

**Purpose:**

Dysregulated expression of proteoglycans influences the outcome and progression of numerous cancers. Several studies have investigated the role of individual glypicans in cancer, however, the impact of the whole glypican family of heparan sulfate proteoglycans on prognosis of a large patient cohort of breast cancer patients has not yet been investigated. In the present study, our aim was to investigate the prognostic power of the glypicans in breast cancer patients.

**Methods:**

We used a public database including both gene expression data and survival information for 3951 breast cancer patients to determine the prognostic value of glypicans on relapse-free survival using Cox regression analysis. Moreover, we performed quantitative Real-Time PCR to determine glypican gene expression levels in seven representative breast cancer cell lines.

**Results:**

We found that high GPC3 levels were associated with a better prognosis in overall breast cancer patients. When stratified by hormone receptor status, we found that in worse prognosis subtypes low GPC1 levels correlate with a longer relapse-free survival, and in more favorable subtypes low GPC6 was associated with longer survival.

**Conclusion:**

Our study concludes that glypicans could act as subtype-specific biomarkers for the prognosis of breast cancer patients and sparks hope for future research on glypicans possibly eventually providing targets for the treatment of the disease.

**Supplementary Information:**

The online version contains supplementary material available at 10.1007/s00432-021-03597-4.

## Introduction

Breast cancer has a high global impact on female health and is the most commonly diagnosed cancer in women worldwide, contributing to 24.2% of all cancers in women. This means that every fourth cancer diagnosis in females is breast cancer (IARC 2018). Even though the prognosis is relatively favorable, it accounts for 15% of cancer deaths in women worldwide and is thus the leading cause of cancer-related mortality (IARC 2018).

Tumors are highly heterogenic and show a large variation of gene expression patterns (Zhang et al. 2018b). Clinically, this heterogeneous disease can be categorized into three different major subtypes. The expression of molecular markers for estrogen receptors (ER) or progesterone receptors (PR) and human epidermal growth factor 2 (HER2) are used as classification factors and play an essential role in the treatment of patients with invasive breast cancer (Duffy et al. 2017). The clinical subtypes are hormone receptor positive/HER2-negative (70% of patients), HER2-positive (15–20%) and triple-negative (TNBC) (lacking all molecular markers, 15%) (Waks and Winer 2019). Those three subtypes have distinct risk profiles, prognoses and treatment regimens (Cheang et al. 2009).

When it is detected early, breast cancer is a curable disease whereas a late diagnosis in an invasive stage is associated with a less favourable outcome. The access to screening methods, early diagnosis and decent quality medical and surgical therapies can be a matter of life and death (Becker 2015).

The major factors causing breast cancer-related deaths are metastasis and uncontrolled proliferation of cancer cells. An important molecular mechanism enabling breast cancer cells to metastasize is the activation of a latent program called epithelial–mesenchymal transition which causes epithelial cancer cells to express mesenchymal-like traits (Castillo et al. 2016). Further mechanisms include aberrant expression of chemokine receptors, enhanced expression of matrix-degrading enzymes such as matrix metalloproteinases and heparanase, and altered cytoskeletal function (Chiang and Massagué 2008; Scully et al. 2012). A number of genes and proteins are discussed with regards to their influence on metastasis and one group emerging from research is the family of glypicans.

Glypicans belong to a family of glycosylphosphatidylinositol-anchored membrane-bound heparan sulfate proteoglycans (HSPG) (Kaur and Cummings 2019). They are cell surface glycoproteins where heparan sulfate glycosaminoglycan chains are linked to a protein core at a membrane-proximal location. Glypicans can act as co-receptors for multiple signaling molecules known for regulating cell growth, motility and differentiation thus they can control cellular morphology and cellular behavior (Li et al. 2018). Evidence has shown that the specific function of individual glypicans depends on their structure and the growth factors present in the specific cellular environment (Filmus and Capurro 2012). It is, therefore, not surprising that changes in glypican gene expression have been mentioned in various human cancers.

Due to their specific structure, glypicans can interact with different classes of proteins, including, but not limited to, morphogens, growth factors, cytokines, chemokines, ECM proteins and adhesion molecules (Hassan et al. 2021). So far, publications suggest that membrane-attached glypicans mainly function by regulating the signaling of Wnts, Hedgehogs (Hhs), fibroblast growth factors (FGFs) and bone morphogenetic proteins (BMPs) (Filmus et al. 2008). Mostly, the regulatory activity stems from the glypicans’ ability to inhibit or stimulate the growth factor interaction with their signaling receptors (Theocharis et al. 2015).

Glypicans are differentially expressed in several cancers, acting as tumor promoters as well as suppressors in a cancer type-specific manner (Kaur and Cummings 2019). For instance in hepatocellular carcinoma (HCC), an overexpression of glypican-3 increases the migratory ability and invasive capacity of cancer cells by reducing cell adhesion to fibronectin (Kwack et al. 2006) whereas it plays an inhibitory role in breast cancer. Here it reduces cell invasive capability and metastasis and promotes cellular adhesion to fibronectin (Peters et al. 2003).

Based on this evidence we hypothesize that certain glypicans could act as valuable prognostic factors of breast cancer survival. Indeed, individual glypicans have been identified as prognostic factors in oesophageal squamous cell carcinoma (GPC1) (Hara et al. 2016), hepatocellular carcinoma (GPC3) (Zhang et al. 2018a) and ovarian cancer (GPC6) (Karapetsas et al. 2015). However, little is known about a potential prognostic value of glypicans in breast cancer. Since the six members of the glypican family have individual features and functions, we aimed at identifying the prognostic power of specific glypicans for breast cancer. The expression of certain specific glypicans might eventually act as a target for therapy; nevertheless, more research is needed in this field.

In this study, we used an updated version of a previously established database (Györffy et al. 2010; Mihály et al. 2013) incorporating the gene expression data of over three thousand breast cancer patients to determine whether the expression of glypicans has an impact on relapse-free survival (RFS). We then examined seven breast cancer cell lines representative of different molecular subtypes by quantitative real-time PCR (qPCR) with respect to their relative gene expression of glypicans.

Our results suggest a prognostic impact of GPC1, GPC3, GPC6 and potentially GPC4, depending on the hormone and HER2-receptor status of tumors. Thus, taking glypican gene expression into account as a subtype-specific biomarker might help to determine the prognosis of breast cancer. Furthermore, glypicans could be considered as possible treatment targets to prolong the relapse-free survival of breast cancer patients.

## Materials and methods

### Survival analysis

To evaluate the prognostic impact of glypican-1–6 on the survival of breast cancer patients, we initially utilized the Kaplan–Meier Plotter online tool (Györffy et al. 2010). Publicly accessible via kmplot.com, the Kaplan–Meier Plotter is an online database established based on gene expression data from Gene Expression Omnibus (GEO), European Genome-phenome Archive (EGA) and The Cancer Genome Atlas (TCGA). The gene expression data was obtained through microarray analysis of widely used arrays of the GEO database and converted into Kaplan–Meier survival curves. Currently, the database consists of data of 54,000 genes and their effect on survival in 21 cancer types (Nagy et al. 2018). The tool draws Kaplan–Meier survival plots to examine the effect of expression levels of specific genes on the clinical outcome of breast cancer patients (Györffy et al. 2010; Lánczky et al. 2016). For breast cancer, presently this program employs relapse-free survival data from 3951 patients and overall survival data from 1402 patients.

The background database of the Kaplan–Meier Plotter is manually curated and handled by a PostgreSQL server, integrating gene expression and clinical data at the same time. The statistical tool behind the calculations is the *R* statistical environment, in which the package “survival” calculates and plots the curves and the number-at-risk of each group is portrayed underneath the plot (Györffy et al. 2010).

In our study, we differentiated ER status, PR status, HER2 status and lymph node (LN) status and we created the triple-negative subtype by selecting “negative” in the categories of ER status, PR status and HER2 status. The Affymetrix IDs for the genes were 202756_s_at for GPC1, 239422_at for GPC2, 209220_at for GPC3, 204984_at for GPC4, 207174_at for GPC5, 227059_at for GPC6.

### Cell culture

All human breast cancer cell lines were purchased from ATCC/LGC Promochem (Wesel, Germany) between 2015 and 2019. Cell lines were selected to represent three subtypes, namely MCF-7 and T47D for the luminal subtype, MDA-MB-231, -453 and -468 for the basal subtype and BT474 and SKBR3 as representatives of the HER2-positive subtype. The major characteristics of each cell line are presented in Table S1. Cells were routinely controlled for expression of hormone receptors by qPCR, checked weekly for correct morphology by phase-contrast microscopy, and regularly authentified by short tandem repeat (STR) analysis. T47D (CVCL_0553), SKBR3 (CVCL_0033), MDA-MB-453 (CVCL_0418), MDA-MB-468 (CVCL_0419) and MDA-MB-231 (CVCL_0062) cells were maintained in DMEM with 1% glutamine, 10% fetal calf serum (FCS) (Gibco^®^, Thermo Scientific, Germany) and 1% penicillin/streptomycin in a humidified atmosphere of 7.5% CO_2_ at 37 °C. MCF-7 (CVCL_0031) cells were cultured in RPMI containing 10% FCS, 1% glutamine and 1% penicillin/streptomycin and maintained in a humidified atmosphere of 5% CO_2_ at 37 °C. BT-474 cells were cultured in RPMI containing 20% FCS, 1% glutamine and 1% penicillin/streptomycin and 0.01 mg/ml insulin in a humidified atmosphere of 5% CO_2_ at 37 °C. All reagents and chemicals, except for the FCS, were from Sigma-Aldrich Chemie GmbH, Taufkirchen, Germany.

### Quantitative real-time PCR

Total RNA was isolated from the cultured human breast cancer cell lines using the InnuPrep RNAMini Kit (Analytik Jena AG, Jena, Germany) according to the manufacturer’s instructions and then reverse transcribed into cDNA with the High-Capacity cDNA Reverse Transcription Kit (Applied Biosystems, Foster City, CA, USA) employed following the supplier’s instructions.

Quantitative real-time PCR was performed in triplicates for each target gene using a Mastermix from Eurogentec (Eurogentec, Liège, Belgium) and we used the Real-Time PCR System ABI PRISM 7300 Sequence Detection System (Thermo Fisher Scientific, Waltham, USA) to detect the gene expression. Primer sequences were confirmed by using the NCBI BLAST analysis. Primers were obtained from Biolegio (Nijmegen, Netherlands) and are stated in Table S2.

Transcriptional analysis was performed using the 2^−ΔCt^ method and the gene expression results were normalized to the Ct-values of β-ACTIN as internal controls. Melting curve analysis was performed to confirm specific product amplification.

### Protein interaction network analysis (STRING)

We used the online bioinformatic tool STRING v11 (http://string-db.org/) to generate in silico protein interaction networks for the gene products that we analyzed, namely GPC1, GPC2, GPC3, GPC4, GPC5, GPC6. STRING aims to collect, score and integrate all publicly available sources that provide information regarding functional and physical protein–protein interaction and displays these in a computed network. The database implements Gene Ontology (GO) and Kyoto Encyclopedia of Genes and Genomes (KEGG) classification systems, and compliments those with new classification systems based on high-throughput text-mining and hierarchical clustering of the association network itself (Szklarczyk et al. 2019). We selected the results to be predicted with the highest confidence threshold of 0.900, and we allowed all predictive methods, i.e. text-mining, experiments, databases, co-expression, neighborhood, gene fusion, co-occurrence. We selected ten interactors on the first, and ten on the second shell.

### Protein interaction network analysis (MatrixDB)

We used MatrixDB (http://matrixdb.univ-lyon1.fr/), a publicly available interaction database focusing on biomolecular interactions established by extracellular matrix (ECM) proteins and glycosaminoglycans (GAG) to uncover further biomolecular interactors with glypcians. MatrixDB is an active member of the International Molecular Exchange (IMEx) consortium and has adopted the HUPO Proteomics Standards Initiative standards for manual curation of the literature and the exchange of interaction data (Clerc et al. 2019).

We performed the analysis on the four glypicans GPC1, GPC3, GPC4, GPC6 that we showed to be promising in breast cancer prognosis to extend our findings from the STRING analysis with extracellular partners. We conducted an extended a query for “glypican” in the *Biomolecule information* search bar, which presents experimentally proven interactions, and added the shown biomolecules to a network using the website’s *iNavigator.* To maximize the relevance of the proteins shown in the network *w*e only added proteins that are present in breast tissue. In a second step, we queried “glypican” in the *Publications* search bar and added the additional biomolecules to the network. To discriminate interactors with the heparan sulfate component of glypicans, we conducted an additional query for “heparan sulfate” and matched the results in Excel with the glypican-interactors.

### Statistical analysis

For survival analysis, in the R statistical environment, we utilized the Kaplan–Meier Plotter database via the statistical package “survival” to calculate Kaplan–Meier survival curves and the number-at-risk. Furthermore, the hazard ratio (and 95% confidence intervals) and log-rank P were calculated for each gene (Györffy et al. 2010). To determine high and low expression, the median was determined and used as a cut-off to reduce the impact of outliers on the results.

All experiments were performed in triplicate and results are expressed as Mean ± SD. GraphPad Prism 8 (GraphPad Software, San Diego, CA, USA) was used to calculate significant differences by a one-way ANOVA and Tukey’s post hoc test. Significant *P* values are indicated by asterisks: ≤ 0.05 by one asterisk *, ≤ 0.01 by two asterisks ** and ≤ 0.001 by three asterisks ***.

## Results

In our research, we investigated the six members of the glypican family and how their genetic expression is associated with the relapse-free survival (RFS) of breast cancer patients. In general, we looked at data of 3951 breast cancer patients using the tool www.kmplot.com/breastcancer and analyzed the RFS. The clinicopathological data of patients can be seen in Table 1.

**Table 1:** Clinico-pathological characteristics of the patients investigated in the present study (Espinoza-Sánchez et al. 2019)

Parameter	Cohort	Proportion
Array platform	HGU133A	52.10%
	HGU133A plus 2.0	47.90%
ER status	ER-positive	76.40%
	ER-negative	23.65%
HER2 status	HER2-positive	17.70%
	HER2-negative	82.30%
Lymph node status	Node positive	39.20%
	Node negative	60.80%
Grade	Grade 1	14.80%
	Grade 2	42.30%
	Grade 3	42.80%
Molecular subtype (St. Gallen)	TNBC	17.10%
	Luminal A	48.60%
	Luminal B	27.70%
	HER2-positive	6.50%
Molecular subtype (Pietenpol)	Basal-like 1	19.2% (within TNBC)
	Basal-like 2	7.8% (within TNBC)
	Immunomodulatory	23.4% (within TNBC)
	Mesenchymal	18.4% (within TNBC)
	Mesenchymal stem-like	9.2% (within TNBC)
	Luminal androgen-receptor	22% (within TNBC)
Age	Mean	53.6 years
	Median	53 years
	Range	24–93 years
Relapse-free survival	Follow-up time (months)	72.8 ± 46.6
	The proportion of events (relapse)	32%
Overall survival	Follow-up time (months)	84.8 ± 47.8
	The proportion of events (death)	25%

*TNBC *triple-negative breast cancer

To break down our analysis into special patient groups and be able to make statements on a subtype-specific level, we stratified the patient cohort by estrogen receptor (ER) status, progesterone receptor (PR) status, HER2 status, lymph node (LN) status and triple-negative status (ER-negative, PR-negative, HER2-negative).

### In the overall patient cohort, higher expression of GPC1 to GPC4 has a statistically significant impact on prolonged survival

We started our analysis by analyzing a total number of 3951 breast cancer patients for glypicans-1, -3, -4, -5 and a total number of 1764 patients for glypicans 2 and 6. The difference in total numbers is that glypican-2 and -6 probes were only present on HGU133 plus 2.0 arrays, whereas the other glypican members were represented both on HG-U133A and HG-U133 plus 2.0 arrays. That is why in the following when referring to patient numbers, we will distinguish patient numbers for glypicans-1, -3, -4, -5 and glypican-2 and -6.

Table 2 shows relapse-free survival and overall survival data of the full patient cohort for glypicans 1–6 (GPC1–GPC6).

**Table 2:** Prognostic significance of the expression of glypicans on the survival of breast cancer patients

Genes	Relapse free survival (= 3951)		Overall survival (= 1402)	
	HR 95% CI	*P* value	HR 95% CI	*P* value
GPC1	0.83 (0.74–0.92)	**0.00055**	1.02 (0.82–1.26)	0.88
GPC2	0.83 (0.71–0.97)	**0.022**	1.17 (0.86–1.6)	0.32
GPC3	0.82 (0.73–0.91)	**0.00029**	0.81 (0.64–1)	0.052
GPC4	0.82 (0.74–0.92)	**0.00039**	0.88 (0.71–1.09)	0.253
GPC5	0.96 (0.86–1.07)	0.45	1.11 (0.89–1.37)	0.36
GPC6	1.1 (0.95–1.29)	0.21	1 (0.73–1.36)	0.98

Bold typing of *P* values indicates a significance level *P* < 0.05

*HR *Hazard ratio, *CI *confidence interval

**Fig. 1:**
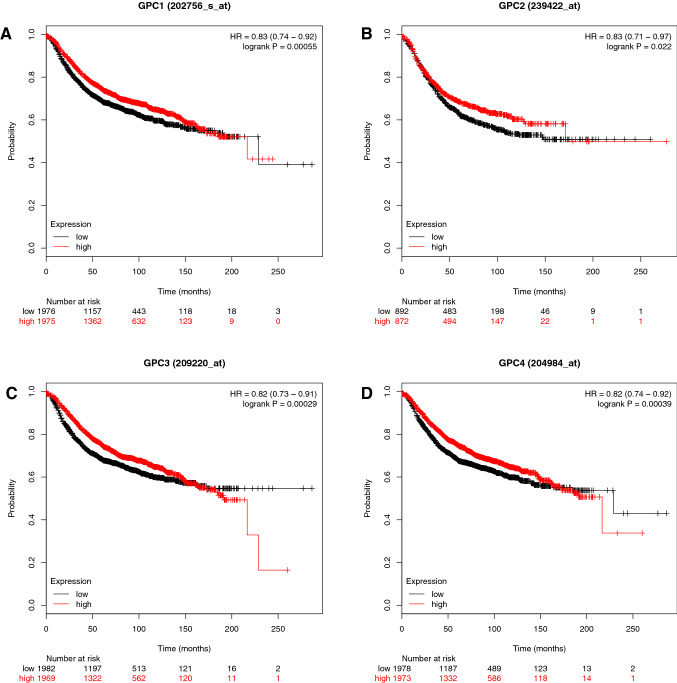
The prognostic value of the gene expression of glypicans. Kaplan–Meier relapse-free survival curves for all 3951 breast cancer patients **a** GPC1, **b** GPC2, **c** GPC3, **d** GPC4. Log-rank *P* values and hazard ratios (HRs; 95% confidence interval in parentheses) are shown. The desired Affymetrix IDs are: 202756_s_at, 239422_at, 209220_at, 204984_at. Discrimination into a high and low expression via using the median as a cut-off

Figure 1 shows that a high expression of the respectively analyzed glypican leads to longer RFS. For GPC1, the *P* value is 0.00055 and HR is 0.83 (A), for GPC2 the *P* value is 0.022 and HR 0.83 (B), for GPC3 the *P* value is 0.00029 and HR 0.82 (C) and for GPC4 the *P* value is 0.00039 and HR 0.82 (D).

### In ER-negative breast cancer, low expression of GPC1 and GPC4 is associated with longer RFS

**Fig. 2:**
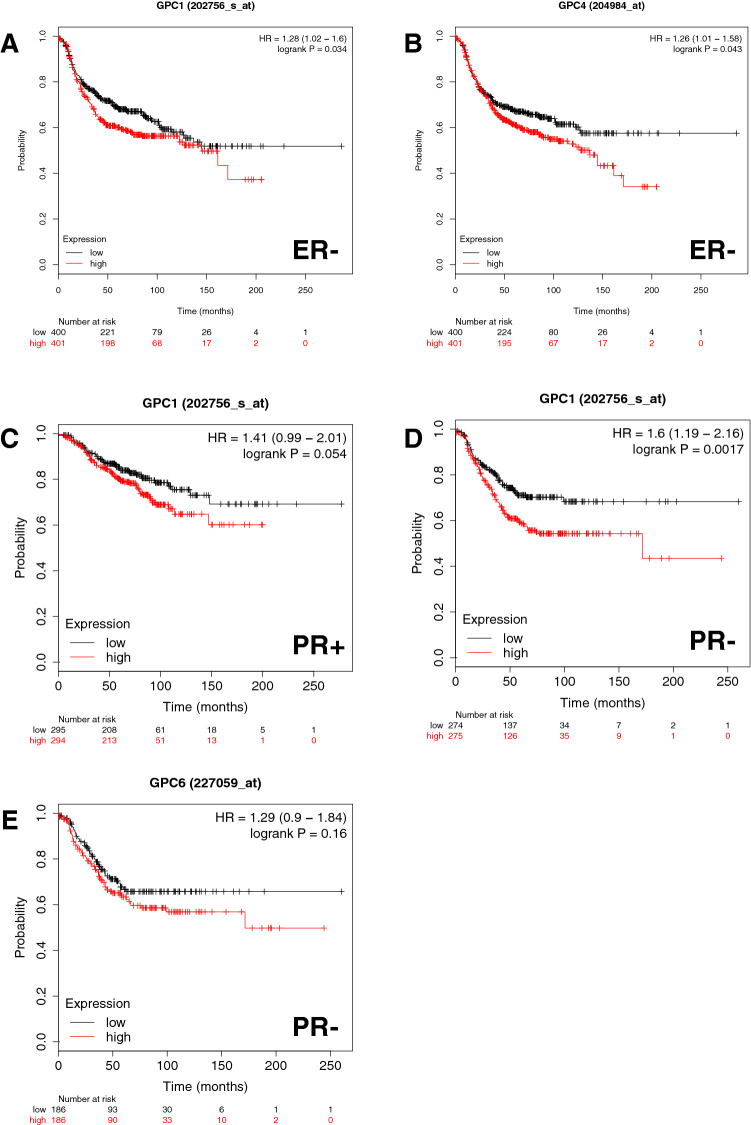
The prognostic value of the expression of glypicans in patients with breast cancer stratified by estrogen receptor (ER) status and progesterone receptor (PR) status. Kaplan–Meier relapse-free survival curves are plotted based on **a** ER− for GPC1, **b** ER− for GPC4 **c** PR+ for GPC1, **d** PR− for GPC1 and **e** PR− for GPC6 Log-rank *P* values and hazard ratios (HRs; 95% confidence interval in parentheses) are shown. The desired Affymetrix IDs are: 202756_s_at, 204984_at and 227059_at. Discrimination into high and low expression via using the median as a cut-off

We next wanted to examine the correlation of estrogen-receptor status with the impact glypicans have on RFS. Therefore, we restricted the analysis to ER-positive and ER-negative patients respectively. For GPC1, 3, 4, 5 we looked at 2061 ER-positive patients and 801 ER-negative patients, and for GPC2 and GPC6 there were 762 ER-positive patients and 347 ER-negative patients (Table 3). We found that a low expression of genes for GPC1 and GPC4 is statistically significantly associated with longer RFS in ER-negative breast cancers (Fig. 2). The *P* value for GPC1 is 0.034 and HR is 1.28 (A), and for GPC4 the *P* value is 0.043 and HR is 1.26 (B). In ER-positive breast cancers, we were not able to see the same effect.

**Table 3:** Prognostic significance of the expression of glypicans on the relapse-free survival of breast cancer patients stratified by estrogen receptor (ER) status

Genes	ER status	Relapse free survival		
		Number of cases	HR 95% CI	*P* value
GPC1	Positive	2061	1.07 (0.9–1.25)	0.448
	Negative	801	1.28 (1.02–1.6)	**0.034**
GPC2	Positive	762	0.98 (0.73–1.31)	0.887
	Negative	347	0.96 (0.69–1.34)	0.828
GPC3	Positive	2061	0.95 (0.8–1.11)	0.51
	Negative	801	0.96 (0.76–1.2)	0.69
GPC4	Positive	2061	0.96 (0.81–1.12)	0.58
	Negative	801	1.26 (1.01–1.58)	**0.043**
GPC5	Positive	2061	0.99 (0.84–1.17)	0.91
	Negative	801	1.03 (0.83–1.3)	0.7658
GPC6	Positive	762	1.21 (0.9–1.62)	0.2
	Negative	347	1.11 (0.79–1.54)	0.55

Bold typing of *P* values indicates a significance level *P* < 0.05

*HR *Hazard ratio, *CI *confidence interval

### In HER2-negative breast cancer, low expression of GPC6 is associated with a longer RFS

Since the HER2 status is an important marker, we next examined whether stratifying the breast cancer patients by HER2 status yielded significant effects on RFS. Therefore, we looked at data from 252 HER2-positive and 800 HER2-negative patients for GPC 1, 3, 4, 5 and 150 HER2-positive and 635 HER2-negative patients for GPC2 and GPC6 (Table 4).

**Table 4:** Prognostic significance of the expression of glypicans on the relapse-free survival of breast cancer patients stratified by HER2 status

Genes	HER2 status	Relapse free survival		
		Number of cases	HR 95% CI	*P* value
GPC1	Positive	252	1.51 (0.97–2.35)	0.063
	Negative	800	1.08 (0.83–1.4)	0.58
GPC2	Positive	150	0.79 (0.46–1.37)	0.41
	Negative	635	0.89 (0.66–1.2)	0.44
GPC3	Positive	252	0.78 (0.51–1.21)	0.27
	Negative	800	0.85 (0.66–1.11)	0.24
GPC4	Positive	252	1.44 (0.93–2.23)	0.099
	Negative	800	0.86 (0.66–1.12)	0.27
GPC5	Positive	252	0.76 (0.49–1.18)	0.22
	Negative	800	0.99 (0.76–1.29)	0.95
GPC6	Positive	150	1.11 (0.64–1.9)	0.72
	Negative	635	1.45 (1.08–1.96)	**0.014**

Bold typing of *P* values indicates a significance level *P* < 0.05

*HR *Hazard ratio, *CI *confidence interval

**Fig. 3:**
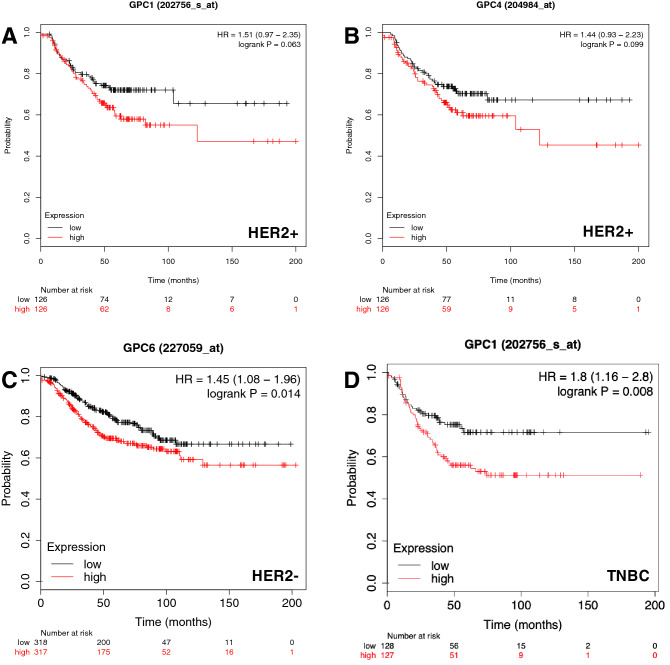
The prognostic value of the expression of glypicans in patients with breast cancer stratified by HER2 status and triple-negative (TNBC) status. Kaplan–Meier relapse-free survival curves are plotted based on **a** HER2+ for GPC1, **b** HER2+ for GPC4, **c** HER2− for GPC6 and **d** TNBC for GPC1. Log-rank *P* values and hazard ratios (HRs; 95% confidence interval in parentheses) are shown. The desired Affymetrix IDs are: 202756_s_at, 204984_at and 227059_at. Discrimination into high and low expression via using the median as a cut-off

The results show that for HER2-negative patients, low expression of GPC6 is associated with a longer RFS, with *P* value 0.014 and HR 1.45 (Fig. 3c).

However, a trend can also be observed for GPC1 (A) and GPC4 (B), although the results were not statistically significant. Interestingly, here the results concern the cohort of HER2-positive patients, as opposed to the results of GPC6 for HER2-negative patients. With a *P* value of 0.063 and HR of 1.51, a low expression of GPC1 is associated with longer RFS in HER2-positive patients. Similarly, low expression of GPC4 is also associated with longer RFS in HER2-positive patients shown by a *P* value of 0.099 and HR of 1.44.

### Low GPC1 expression is correlated with longer RFS in PR-positive and PR-negative breast cancer

We also investigated whether the PR status modifies the effect of the different glypicans on RFS. Therefore, we analyzed data from 589 PR-positive patients and 549 PR-negative patients for GPC 1, 3, 4, 5 and 489 PR-positive and 372 PR-negative patients for GPC2 and GPC6 (Table 5).

**Table 5:** Prognostic significance of the expression of glypicans on the relapse-free survival of breast cancer patients stratified by progesterone receptor (PR) status

Genes	PR Status	Relapse free survival		
		Number of cases	HR 95% CI	*P* value
GPC1	Positive	589	1.41 (0.99–2.01)	**0.054**
	Negative	549	1.6 ( 1.19–2.16)	**0.0017**
GPC2	Positive	489	1.1 (0.75–1.61)	0.63
	Negative	372	0.98 (0.69–1.4)	0.92
GPC3	Positive	589	0.94 (0.66–1.33)	0.72
	Negative	549	1 (0.75–1.34)	1
GPC4	Positive	589	1.11 (0.78–1.57)	0.55
	Negative	549	1.05 (0.79–1.41)	0.74
GPC5	Positive	589	1.18 (0.83–1.66)	0.36
	Negative	549	0.99 (0.74–1.32)	0.93
GPC6	Positive	489	1.19 (0.81–1.74)	0.37
	Negative	372	1.29 (0.9–1.84)	0.16

Bold typing of *P* values indicates a significance level *P* < 0.05

*HR *Hazard ratio, *CI *confidence interval

We found out that low expression of GPC1 is correlated with longer RFS, regardless of PR status (Fig. 2). For PR-positive status the *P* value is 0.054 and HR is 1.41 (A) and for PR-negative status the *P* value is 0.0017 and HR is 1.6 (B).

Additionally, a noteworthy trend can be seen for GPC6 (C), suggesting that low expression of the gene is associated with longer RFS for PR negative patients. However, this result is not statistically significant with a *P* value of 0.16 and a HR of 1.26.

### In triple-negative breast cancer, a low GPC1 expression is associated with longer RFS

Triple-negative breast cancer, also called basal breast cancer, has the worst prognosis out of the intrinsic subtypes (Waks and Winer 2019), which is why it is particularly interesting to find predictors of RFS here, and possibly base treatments on them. To generate triple-negative breast cancer in the KM plot, we selected ER-negative, PR-negative and HER2-negative as restrictions in the tool. We analyzed RFS data in 255 patients for GPC1, 3, 4, 5, and 161 patients for GPC2 and GPC6 respectively, all classified by the basal or triple-negative subtype (Table 6). The results for GPC1 were significant with a *P* value of 0.008 and HR of 1.8 (Fig. 3), meaning that a low expression of the GPC1 gene is associated with a longer RFS.

**Table 6:** Prognostic significance of the expression of glypicans on the relapse-free survival of breast cancer patients stratified by triple-negative status

Genes	Triple status (ER/PR/HER2)	Relapse free survival		
		Number of cases	HR 95% CI	*P* value
GPC1	Negative	255	1.8 (1.16–2.8)	**0.008**
GPC2	Negative	161	1.38 (0.8–2.39)	0.24
GPC3	Negative	255	1.05 (0.68–1.6)	0.84
GPC4	Negative	255	1.06 (0.7–1.62)	0.78
GPC5	Negative	255	1.1 (0.72–1.69)	0.65
GPC6	Negative	161	1.47 (0.85–2.56)	0.16

Bold typing of *P* values indicates a significance level *P* < 0.05

*HR *Hazard ratio, *CI *confidence interval

### The impact of glypican expression on the prognosis of breast cancer patients is independent of lymph node status

To examine whether the impact of genetic expression of glypicans on RFS is dependent on or modified by lymph node status, we ran a KMplot analysis on 1133 patients with positive lymph node status and 2020 patients with LN-negative status for GPC 1, 3, 4, 5 and, furthermore, 724 LN-positive and 496 LN-negative patients for GPC2 and GPC6.

When stratified by lymph node status, there are no statistically significant findings from any of the glypicans influencing relapse-free survival. We can conclude here, that the prognostic impact of glypican expression is independent of the lymph node status (Table S4).

### Glypican-1 and -4 are the most highly expressed glypicans in a panel of representative human breast cancer cell lines

Tumors are embedded within their tumor microenvironment, which consists of fibroblasts, smooth muscle fibers, adipocytes, endothelial cells and immune cells (Kaur and Cummings 2019). The data from the patient samples were generated from whole tumors, so automatically the genetic analysis also detected genes that were not located in the tumor cells themselves but in the microenvironmental cells. Therefore, in an approach to validate our findings from the tumor samples, we additionally investigated human breast cancer cell lines representative of different molecular subtypes. Those cell lines only consist of pure tumor cells and thus no gene expression data from the tumor microenvironment will be taken into account, reducing the bias of results.

We performed quantitative real-time PCR (qPCR) to measure the relative gene expression of the respective glypicans, GPC1, GPC2, GPC3, GPC4, GPC5 and GPC6 in seven breast cancer cell lines; MCF-7 and T47D as representatives of the luminal molecular subtype, SKBR3 and BT-474 as representatives of the HER2 subtype and MDA-MB-231, MDA-MB-453, MDA-MB-468 as representatives of the basal subtype. ﻿

We evaluated the qPCR results from two dimensions; first, we analyzed the relative gene expression of different glypicans within a specific cell line, and second, we analyzed a specific glypican and looked at its relative gene expression among the cell lines.

We expected that cell lines belonging to the same intrinsic subtype have an analogous gene expression of glypicans. However, this was only the case in some exceptions, so overall we cannot conclude that breast cancer cell lines of the same intrinsic subtype show a similar glypican gene expression.

**Fig. 4:**
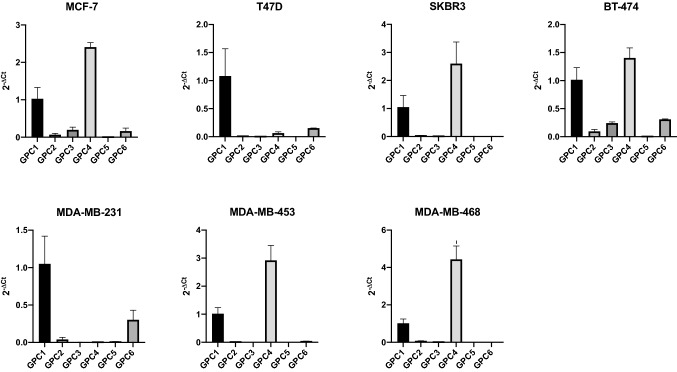
Presentation of relative mRNA expression of glypican genes within a specific cell line. Gene expression of GPC1, GPC2, GPC3, GPC4, GPC5 and GPC6 was quantified by qPCR in 7 breast cancer cell lines representative of the luminal (MCF-7 and T47D), basal (MDA-MB-231, -453 and -468) and HER2-positive (BT474 and SKBR3) breast cancer subtypes. Gene expression was normalized to the expression of β-actin and is represented by 2^−ΔCt^. Error bars indicate the mean and standard deviation (SD) from three individual experiments conducted in triplicates

Both HER2 breast cancer cell lines, SKBR3 and BT-474, show similar relative gene expression levels of GPC1 and GPC4. The basal breast cancer cell lines MDA-MB-453 and MDA-MB-468 show a similar glypican gene expression pattern, with high expression of GPC1 and GPC4 and MDA-MB-231 shows a high expression of GPC1 and GPC6 (Fig. 4). In general, Fig. 4 shows GPC1 to be expressed in all the seven cell lines and GPC4 is highly expressed in five out of the seven cell lines. Four cell lines show GPC6 expression, namely MCF-7, T47D, BT-474 and MDA-MB-231. Strikingly, the relative expression of GPC2 and GPC5 is minimal in all of the cell lines.

### Glypican-3 is down-regulated in breast cancer cells of the basal subtype

For GPC1 we noted that there was a high level of expression in T47D, a cell line of the luminal subtype, significantly different from all other cell lines. There is a very low GPC3 expression in cell lines of the basal subtype, MDA-MB-231, MDA-MB-453 and MDA-MB-468. Those are significantly different from the other cell lines.

**Fig. 5:**
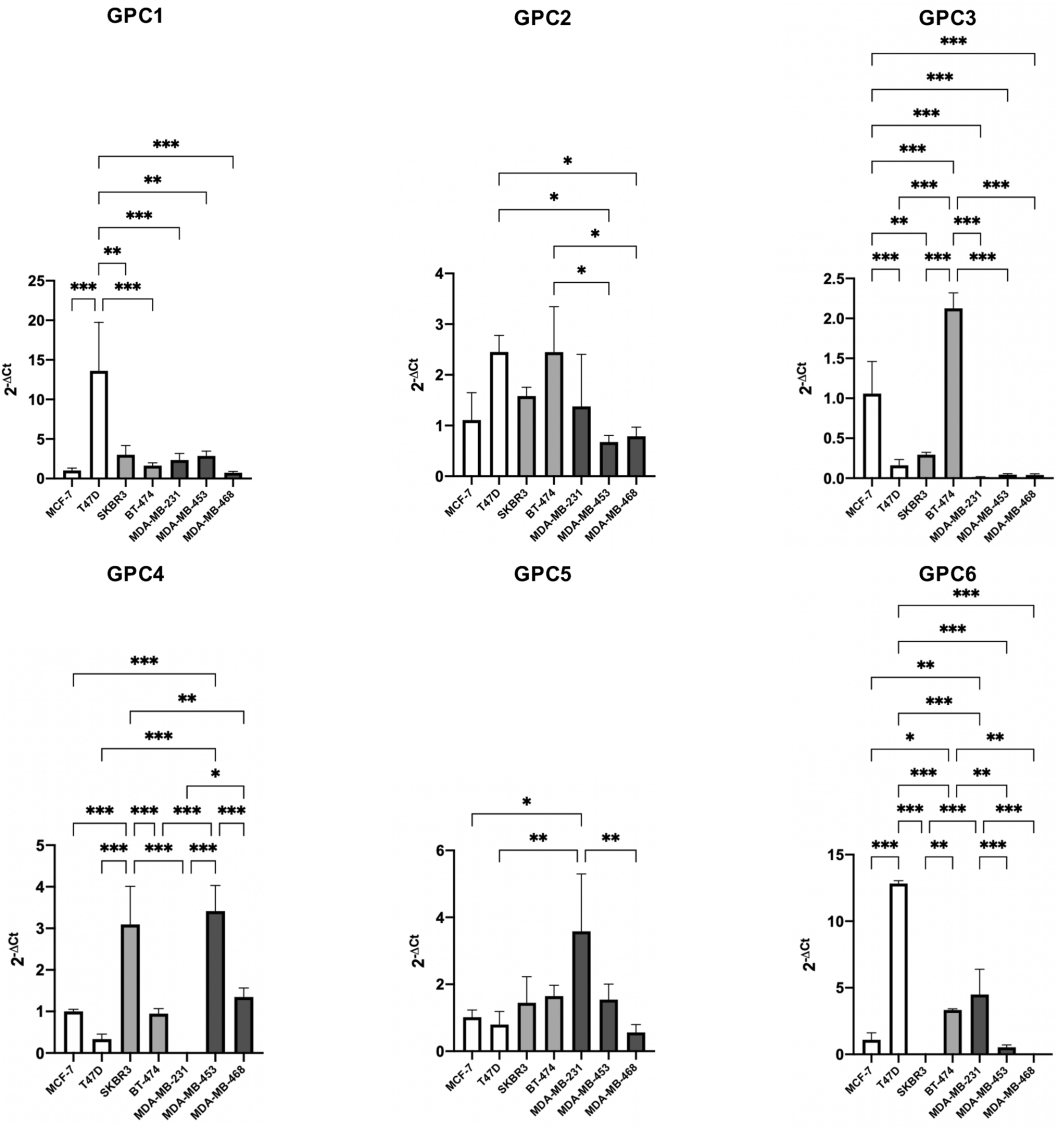
Presentation of relative mRNA expression of a specific glypican gene in seven breast cancer cell lines. Expression of the genes GPC1, GPC2, GPC3, GPC4, GPC5 and GPC6 was quantified by qPCR in 7 breast cancer cell lines representative of the luminal (MCF-7 and T47D), basal (MDA-MB-231, -453 and -468) and HER2-positive (BT474 and SKBR3) breast cancer subtypes. Gene expression was normalized to the expression of β-actin and is represented by 2^−ΔCt^. Error bars indicate the mean and standard deviation (SD) from three individual experiments conducted in triplicates. To test for the difference between mean gene expressions in the different breast cancer cell lines, one-way ANOVA was employed, followed by the Tukey post hoc test to correct for multiple comparisons. *P < 0.05, **P < 0.01 and ***P < 0.001

GPC4 is more abundant in the more invasive cell lines (HER2 and basal subtype) with the exception of MDA-MB-231. We noticed a very high GPC6 expression in the T47D cell line of the luminal subtype. For GPC2 and GPC5 there were no statistically significant differences between expression in the cell lines. However, also in this dimension of looking at the results, the gene expression largely varies between the cell lines, even between cell lines that belong to the same intrinsic subtype (Fig. 5).

### Functional enrichment analysis reveals interactions of glypicans with syndecans and the hedgehog and growth factor signaling pathways

As the next step in our analysis, we used the online bioinformatic tool STRING to create a functional interaction network of the six glypicans, GPC1, GPC2, GPC3, GPC4, GPC5, GPC6, portraying interactions between the glypicans themselves plus two shells of interactors.

**Fig. 6:**
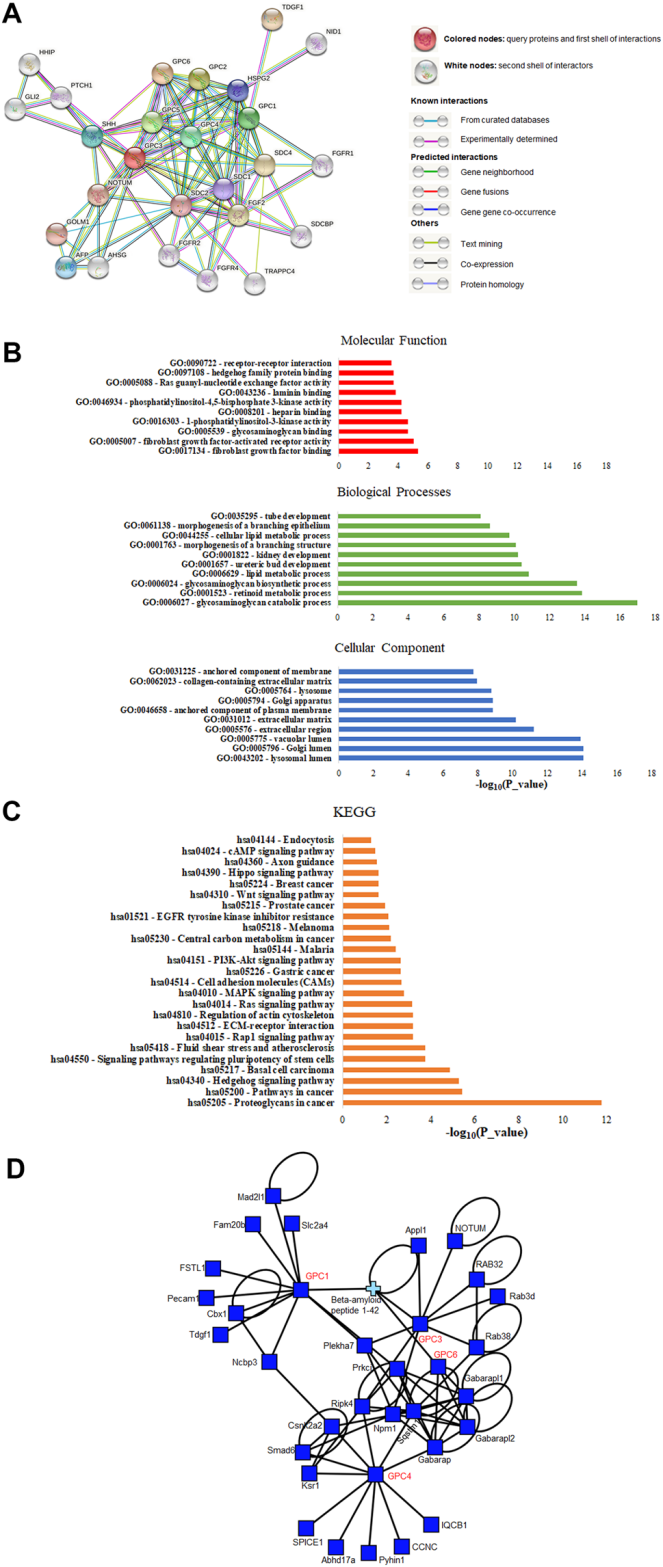
The network of glypicans and their interactors. **a** String database output depicting functional and physical interactors of the glypican family, GPC1, GPC2, GPC3, GPC4, GPC5 and GPC6 obtained from http://string-db.org/. The analyzed proteins are highlighted in red boxes. Highest confidence threshold of 0.900. **b** Gene ontology (GO) analysis of the glypicans. The 10 most significantly (FDR < 0.05) enriched GO terms in molecular function (red), cellular component (blue), and biological process (green) branches are presented. **c** KEGG pathway analysis. All the adjusted statistically significant values of the terms were negative 10-base log-transformed. **d** Protein–protein interaction network of prognostically relevant glypicans built using the iNavigator tool of the MatrixDB interaction database (Clerk et al. 2019)

First, we could confirm our expectations, that all six glypicans are highly interconnected, which can be seen in Fig. 6a by the amount of edges connecting the GPC-nodes. Second, interactions with NOTUM, SHH and PTCH1 were observed. This comes as no surprise, since these proteins are all involved in the molecular functioning of glypicans. NOTUM, a palmitoleoyl-protein carboxylesterase, acts as a key negative regulator of the Wnt signaling pathway (Traister et al. 2008) and cleaves the glypican GPI anchor to release it from the cell surface. Sonic hedgehog (SHH) release is controlled by glypicans, which stimulate its proteolytic processing into bioactive sonic hedgehog. Genes related to the hedgehog pathway have been shown to be associated with better survival of breast cancer patients (Kuehn et al. 2021). Protein patched homolog 1 (PTCH1) serves as a receptor for SHH and associates with the smoothened protein (SMO) to transduce the hedgehog’s protein signals (Filmus and Capurro 2014). In addition to that, syndecans (SDC1, SDC2, SDC4) are part of the network and are highly interconnected with the glypicans. Syndecans and glypicans collaborate closely but are not interchangeable, as has been shown by Ding et al. (2005). The authors presented that the FGF2-growth factor-induced shedding of syndecan-1 from the cell membrane makes the mitogenic response of pancreatic cancer cells glypican-1 dependent, which in turn cannot be compensated for by syndecan-1 (Ding et al. 2005).

Finally, we can see the aforementioned growth factor FGF2 and FGFR (1, 2 and 4) which play an important role in cell survival, cell differentiation and cell migration (Huang et al. 2017).

Figure 6 also shows the Gene Ontology analysis of molecular function, biological processes and cellular components of the proteins we investigated. Especially the molecular function terms were in line with our analysis portraying the role in tumorigenesis by binding to growth factor receptors, the signaling pathways and proliferation and adhesion functions. Basement membrane components are also visible which may be relevant for metastasis. The KEGG analysis revealed enriched pathways linked to hedgehog signaling pathway, proteoglycans in cancer, ECM-receptor interaction, Wnt signaling pathway and breast cancer, mainly underlining the signaling pathways for breast cancer progression.

To expand our findings from the STRING protein–protein interaction network, we queried the extracellular protein database MatrixDB. We looked for experimentally supported interactions with GPC1, GPC3, GPC4, and GPC6 as these are the glypicans we found to have the highest impact on the prognosis of breast cancer patients. NOTUM is also a partner in this network, but a number of relevant new partners came to light (Fig. 6d, Table S5). Platelet endothelial cell adhesion molecule 1 (Pecam1) is an angiogenesis marker that has been suggested to assess the influence of microvessels and angiogenesis on the clinical outcome of breast cancer patients (Martin et al. 2005). Furthermore we established Mothers against decapentaplegic homolog 6 (Smad6) as an interactor of glypicans. ER-negative breast cancer patients who express high levels of the gene were shown to have a poor distant-metastasis free survival (De Boeck et al. 2016). Our MatrixDB analysis also showed tumor-derived growth factor 1 (Tdgf-1), an embryonic stem cell marker, as a partner of glypicans. Tdgf-1, also known as Cripto-1, has been investigated as a potential treatment for triple-negative breast cancer in a study by Castro et al. who found that Cripto-1 knockout in a mouse model has reduced tumor growth and lung metastasis (Castro et al. 2015). Another interesting interaction partner was Casein kinase II subunit alpha (Csnk2a2), a subunit of casein kinase 2 (CK2), which is discussed to reduce prosurvival signaling cascades and cell survival in breast cancer cells (Gray et al. 2014). The authors have shown that the breast cancer cells lines MDA-MB-231 and MCF-7 portrayed increased levels of CK2, and they found that when the cell lines were treated with CX-4945, a CK2-inhibitor, cells became apoptotic and in MDA-MB-231 metastatic behavior was reduced.

In addition to the experimentally proven interactions with glypicans, we queried MatrixDB for interactions based on literature findings. A number of additional partners were found and added to the network (Table S5). For example, we found the extracellular ligands Wnt and the Frizzled receptors they bind to interact with glypicans. This has been investigated by Okolicsanyi et al. who examined the expression profile of HSPG core proteins glypicans and found that cell motility and tumorigeneity are mediated by the interaction with members of the Wnt pathway (Okolicsanyi et al. 2014).

To discriminate whether the identified partners interact with the heparan sulfate part of the glypicans rather than their glycosaminoglycan chains, we determined the interactors of heparan sulfates in MatrixDB and matched those with the glypican-interactors. Out of three matched partners (fibronectin, apolipoprotein E, and beta-amyloid peptide 1–42), fibronectin is highly relevant in the context of our study, since a high expression of this interstitial matrix molecule and integrin ligand in primary tumors resulted in decreased survival of breast cancer patients (Shinde et al. 2018).

## Discussion

In this study, our aim was to identify whether any member of the glypican family, glypican-1 to glypican-6, has a prognostic impact on the survival of breast cancer patients and could potentially act as therapeutic targets in the battle against the most common cancer in women. To approach this research question, firstly we utilized a large cohort of breast cancer patients to analyze the impact GPC1 to GPC6 have on their relapse-free survival. The database contains data of 3951 patients and as such is a preferred way to start analysis into the effect of genes on the survival of a large cohort of cancer patients (Györffy et al. 2010).

**Fig. 7:**
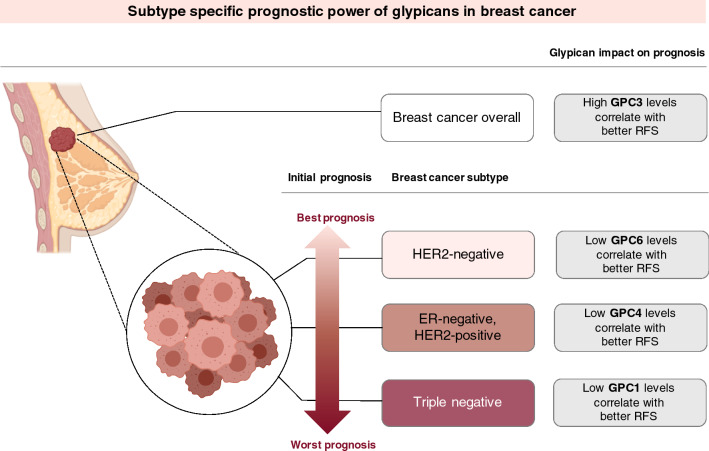
Overview of subtype-specific prognostic power of glypicans in breast cancer. Summary of key results of the present study. For overall breast cancer patients, that have not been classified by receptor status, higher levels of GPC3 are associated with longer RFS. If patients are stratified by receptor status, GPC6 is a prognostic marker for subtypes that have an initially better prognosis. Low GPC6 levels are correlated with a longer RFS. GPC1 is a prognostic marker for initially worse prognosis subtypes, and low levels of the glypican are associated with longer RFS. Adapted from “Intrinsic and Molecular Subtypes of Breast Cancer”, by BioRender.com (2020) and created in PowerPoint. Retrieved from https://app.biorender.com/biorender-templates

We then analyzed seven breast cancer cell lines by quantitative real-time PCR to determine their relative gene expression levels of glypicans. We undertook this step of analysis in a quest to establish whether our results for step 1 of this study actually stem from the tumor cells themselves, or whether the tumor microenvironment contained the genes affecting the results. The key findings from this study are summarized in Fig. 7. We found that high levels of GPC3 expression are associated with a longer survival in breast cancer patients. When differentiated by receptor status, we found that worse prognosis subtypes had a longer relapse-free survival with low GPC1 levels, whereas in subtypes with a better prognosis, low GPC6 levels were associated with longer survival. Notably, the expression levels of two glypicans not associated with breast cancer prognosis (GPC2 and GPC5) were very low in the breast cancer cell lines studied, which may be linked to the lack of a prognostic value. In addition, partially diverging functional roles have been assigned to different members of the glypican family (discussed in Karamanos et al. 2018): while GPC5 stimulates cell proliferation in rhabdomyosarcoma by enhancing Shh binding, GPC3 competes with the receptor for ligand binding via a mechanism that depends on proteolytic processing. Moreover, in Simpson–Golabi–Behmel syndrome, a human genetic disease characterized by overgrowth (Pilia et al. 1996), where aberrant function of GPC3 affects Hh signaling (Capurro et al. 2008), none of the other glypican family members can substitute for GPC3 (Yoneda et al. 2012). Different biological functions of individual glypicans such as this impact on hedgehog signaling may have contributed to their distinct prognostic roles in breast cancer.

### Glypican-1

The expression of GPC1 has been linked to the pathogenesis of numerous tumor entities including pancreatic cancer (Aikawa et al. 2008), esophageal squamous cell carcinoma (ESCC) (Hara et al. 2016), and glioma (Su et al. 2006). In breast cancer, GPC1 is overexpressed and modulates heparin-binding growth factors and FGF2, thus suggesting a role in breast cancer progression (Matsuda et al. 2001). Nevertheless, here we have to note that in a more recent study by Fernández-Vega et al. no elevated levels of GPC1 were detected in 23 breast tumor samples (Fernández-Vega et al. 2013).

Our study has shown that in unclassified breast cancer, high GPC1 levels lead to a longer RFS compared to those with lower levels. However, if we look at breast cancer stratified by receptor status, we find that in ER-negative breast cancer, low expression of GPC1 is associated with longer RFS in patients. Furthermore, in HER2-positive patients, low GPC1 levels also lead to longer RFS. PR-positive as well as PR-negative patients show a longer RFS with low expressions of GPC1. In triple-negative breast cancer, low expression of GPC1 showed a positive effect on RFS. Our cell line data show that GPC1 is expressed in all the seven cell lines we examined, with T47D, a cell line of the luminal subtype, showing the highest level of relative gene expression. This strengthens our findings on the prognostic value of GPC1, since GPC1 is not only present in the tumor microenvironment, but can be found in tumor cells themselves (Fig. 5).

The removal of GPC1 from cancer cells which show higher gene expression levels than healthy cells could make these cells insensitive to a number of growth factors. More precisely this has been shown for GPC1 in pancreatic cancer cells, where syndecan-1 and GPC1 are both required for FGF2-growth factor response leading to metastasis and shedding of GPC1 cannot be compensated for by higher levels of SDC1 (Ding et al. 2005). This was also suggested in a study by Yoneda et al., where the reduction of GPC1 protein level abrogated mitogenic response of cells to heparin-binding growth factors, as well as heregulin α, heregulin β, and hepatocyte growth factor (HGF) (Yoneda et al. 2012). In breast cancer cell lines, silencing of GPC1 has been examined by Matsuda et al. (2001). By means of using a GPC1 antisense construct, the authors showed that a reduction of GPC1 expression reduced the mitogenic response to several heparin-binding growth factors (Matsuda et al. 2001). Furthermore, GPC1 knockdown by shRNA has been shown to have markedly decreased proliferation in tumor cells in the presence of the a3(V) chain (Huang et al. 2017).

Strikingly, our results show that for the relatively less favorable subtypes in which prognosis is worse, low levels of GPC1 are associated with a longer RFS. This lets us make two conclusions: First, we suggest that if the clinicopathological analysis of receptor status results in hormone receptor negative or HER2-positive breast cancer, the next step to determine the prognosis could be to conduct a further genetic analysis of the tumor and determine the level of GPC1 expression. This idea would be analogous to genetic profiling tests such as Oncotype DX^®^ which specifically looks at hormone receptor positive and HER2-negative patients and determines whether they would benefit from chemotherapy (Siow et al. 2018). For low levels of GPC1, RFS is longer and for high levels it is shorter. The qualities of GPC1 as a potential novel biomarker for prognosis of breast cancer, especially triple-negative breast cancer, were also suggested in a recent study on exosomal proteomes (Risha et al. 2020).

### Glypican-3

﻿GPC3 is the most studied glypican with regards to cancer. After birth, most healthy tissues do not express much GPC3, although some regenerating tissues do (Moek et al. 2018). However, in tissues that only express GPC3 in the embryonic stage, it tends to reappear when there is a malignant transformation into a tumor whereas GPC3-heavy tissues in adults show reduced levels of GPC3 in a malignant situation (Castillo et al. 2016). Depending on the malignancy, GPC3 can have different functions, such as either inhibiting or activating the Wnt signaling pathway (Capurro et al. 2005; Stigliano et al. 2009).

Our survival analysis has shown that high gene expression levels of GPC3 significantly lead to a longer RFS in breast cancer patients overall. When stratified by receptor status, we did not obtain further significant results.

The positive effect on survival correlates with findings from a number of authors who found that GPC3 has a protective role against human breast cancer progression and is in turn downregulated in breast cancer (Fernández-Vega et al. 2013). Two studies have shown that in breast cancer, GPC3 is downregulated by means of hypermethylation of the GPC3-promoter (Xiang et al. 2001; Yan et al. 2001) and Yan et al. have described that the silencing of GPC3 by means of promoter hypermethylation is more predominant in hormone-receptor negative patients, implying that GPC3 is expressed less in those patients.

However, contradicting this, in 2015 Castillo et al. proposed that neither GPC3 mRNA nor protein expression were established with any of the established prognosis markers for their sample of Brazilian and Argentine patients, thus suggesting GPC3 as an independent biomarker (Castillo et al. 2015). The study included n = 121 breast tumor tissues investigated by qPCR, and in the publication the authors themselves suggested conducting research on a larger patient cohort. This is what we have done in the present study, looking at gene expression data of almost four thousand breast cancer patients.

It was a striking finding from our qPCR experiments that the relative expression of GPC3 was very low and almost undetectable in the triple-negative cell lines, which also have the worst prognosis (Fig. 5). In the more favorable subtypes, we see a higher GPC3 expression. Since GPC3 is a glypican where high levels of gene expression are favorable for survival as it reduces the metastatic abilities, it is plausible that there is a low expression in the metastatic subtypes. This is a strong indication for the idea to conduct research on the possible induction of expression of GPC3 in breast cancer patients with the triple-negative subtype, as possibly metastasis of the breast cancer cells can be reduced.

Castillo et al. have suggested GPC3 as a metastatic suppressor since they found that GPC3 has an influence in a number of steps along the metastasis cascade where it induces mesenchymal–epithelial-transition (Castillo et al. 2016). More detailed, via blocking autocrine and paracrine canonical Wnt activities, it causes breast cancer cells to modify their cytoskeleton organization, decrease their ability to migrate and clone, to be more susceptible to apoptosis and to be less invasive (Castillo et al. 2016; Fernández et al. 2018). Wnt has been shown to be aberrantly activated in cancers (Krishnamurthy and Kurzrock 2018).

Since metastasis is the main factor influencing patient survival, discovering molecules with the ability to revert epithelial–mesenchymal transition or to promote mesenchymal–epithelial transition is essential for developing novel cancer treatments (Castillo et al. 2016). Further research into the potency of GPC3 as an anticancer therapeutic would definitely be a valuable step in the battle to fight breast cancer. As a step in the nearer future in line with our findings we propose GPC3 as a marker to identify the prognosis of breast cancer patients.

### Glypican-4

GPC4 is one of the lesser-studied members of the glypican family. A recent study by Munir et al. suggested that downregulation of GPC4 could possibly increase cell migration, invasion and proliferative activities in breast cancer (Munir et al. 2020). Interestingly, the authors showed that knock-down of GPC4 expression in MCF-7 cells using siRNA resulted in more migration activity of the cells.

In the present study, we have found that for overall unclassified breast cancer, high levels of GPC4 showed a longer RFS. In contrast to this, the subtype-specific results for the less favorable clinicopathological subtypes, ER-negative and HER2-positive, showed low GPC4 levels to lead to a longer RFS. It is interesting that for the overall unclassified breast cancer, our results were opposite. Therefore, we assume GPC4 to be a subtype-specific biomarker. Our in vitro cell line data show a high relative gene expression of GPC4 in the HER2 breast cancer cell lines, SKBR3 and BT-474. Furthermore, GPC4 is highly expressed in the metastatic cell lines MDA-MB-453 and MDA-MB-468. This shows GPC4 to be expressed relatively more amongst the less favorable clinicopathological subtypes. However, we cannot infer that it is exclusively expressed in worse subtypes, since MCF-7-cells also show a high relative gene expression (Fig. 5). Together with the results from our survival analysis, we can conclude patients of those subtypes have a better prognosis with low levels of GPC4. Due to the partially contradicting results with the study by Munir et al. (2020), we would encourage future research to look at this very relevant topic in more detail and to potentially investigate whether the presence or absence of hormone receptors has further effects on the capability of glypicans to perform their function.

### Glypican-6

GPC6 has been shown to stimulate Hh signaling by binding to Hh and Patched 1 (Ptc1) through its core protein and glycosaminoglycan chains (Capurro et al. 2017), which is also visible for PTCH1 as part of the functional enrichment analysis in Fig. 6.

A study has shown that GPC6 promotes the invasive migratory capacity of breast cancer cells through non-canonical Wnt5a signaling, where NFAT signaling promotes GPC6 expression in the cells (Yiu et al. 2011). The same study also postulates that silencing GPC6 with shRNA (small-hairpin RNA) blocks this phenotype, thus not expressing the invasive migration. Conversely this means that via the expression of GPC6, NFAT induces a transcriptional program promoting metastatic invasion (Yiu et al. 2011). Silencing GPC6 could potentially be a target of therapeutics. The context and cancer-specific characteristics of the different glypicans yet again become obvious when we look at the fact that in ovarian cancer, high GPC6 expression correlated with increased overall survival (Kaur and Cummings 2019). It has to be noted that a more recent study has found no difference in the glypican-6 mRNA levels in invasive breast cancer vs that of normal mammary tissues (Fernández-Vega et al. 2013). However, the sample size is considerably small with only 23 tumors analyzed.

We have found in our study, that for HER2-negative patients, low GPC6 expression is associated with a longer RFS. Thus we suggest that once the histological subtype is determined, a next step could be to look at GPC6 expression to determine the impact on prognosis. High GPC6 levels would be expected to have a worse prognosis due to the metastatic abilities activated through NFAT (Yiu et al. 2011). A quest for further research into therapeutics focusing on glypicans could be to silence GPC6 as suggested by Yiu et al. (2011) to lower the metastatic ability and thus to improve the prognosis for RFS of breast cancer patients.

In our qPCR analysis, it was shown that the relative gene expression of GPC6 was the highest in T47D cells (Fig. 5), representative for the luminal A subtype. Here we found high GPC6 expression in a cell line with HER2-negative status, which is an indicator that our results are valid and should not only be attributed to the tumor microenvironment.

### Outlook on the potential of therapies based on glypicans

Previously, Espinoza-Sánchez and Götte have discussed that cell surface proteoglycans are attractive targets for cancer and have presented current developments for immunotherapy targeting glypicans in cancer (Espinoza-Sánchez and Götte 2020), where studies and clinical trials have shown great potential. Especially GPC3 has been studied with regards to therapies of HCC where the recombinant fully humanized monoclonal antibody GC33 has shown good tolerance in phase I and II trials (Zhu et al. 2013; Ikeda et al. 2014). Furthermore, GPC3 peptide vaccines have been tested as (adjuvant) HCC therapy where they improved the 1-year recurrence rate of GPC3-positive tumors (Sawada et al. 2016) and an anti GPC-3/CD3 bispecific T cell-redirecting antibody has been investigated for the treatment of solid tumors (Ishiguro et al. 2017). In pediatric tumors, the vaccine has been shown to infiltrate tumor tissue and an antibody has induced regression of solid tumors (Tsuchiya et al. 2018). These results for immunotherapy targeting GPC3 in HCC show, that the glypican family is a promising target for cancer therapies, and studies examining the therapeutic power in breast cancer would be extremely valuable.

## Conclusion

In summary, we have found promising results in our study suggesting the prognostic power of GPC1, GPC3, GPC6 and potentially also GPC4 expression on the survival of breast cancer patients. Precisely, we have found that especially in subtypes with less favorable prognosis, low expression of GPC1 seems to predict longer RFS whereas in the more favorable subtypes, low levels of GPC6 help predict longer RFS. In addition to that, we have found GPC3 to be a powerful predictor for breast cancer patients overall, where high levels of GPC3 lead to longer RFS. As a consequence from our study, we propose the utilization of the glypicans mentioned above as subtype-specific biomarkers. Due to the heterogeneity of the members of the glypican family and the strong evidence of their context-dependent functions (Karamanos et al. 2018), our recommendation focuses on individual glypicans and not the family as a whole.

In the future, GPC1, GPC3, GPC4 and GPC6 might possibly serve as a basis for the medical treatment of breast cancer patients. However, it is arguably optimistic to expect that therapies targeting a single cell surface HSPG alone will provide a novel opportunity for breast cancer treatment since glypicans function alongside other receptors. Nevertheless, the promising data on GPC3 in the context of immunotherapy indicate that further preclinical translational research on glypicans in a breast cancer setting may be worthwhile.

## Supplementary Information

Below is the link to the electronic supplementary material.

Supplementary file1 (DOCX 29 KB)

## Data Availability

The datasets generated and analysed during the first part of the current study, the survival analysis, are available in the Kaplan–Meier Plotter database https://kmplot.com/analysis/. The datasets generated and analysed during the second part of the current study, the cell line data, are available from the corresponding author on reasonable request.

## Abbreviations

EGA: European Genome-phenome Archive
ER: Estrogen receptor
FGF: Fibroblast growth factor
GEO: Gene Expression Omnibus
GPC: Glypican
GPI: Glycosylphosphatidylinositol
HCC: Hepatocellular carcinoma
Hh: Hedgehog
HER2: Human epidermal growth factor receptor 2
HSPG: Heparan sulfate proteoglycans
HR: Hazard ratio
IARC: International agency for research on cancer
NFAT: Nuclear factor of activated T-cells
PR: Progesterone receptor
Ptc: Patched
qPCR: Quantitative Real-Time PCR
RFS: Relapse-free survival
SD: Standard deviation
SDC: Syndecan
SHH: Sonic Hedgehog
TCGA: The cancer genome atlas
TNBC: Triple-negative breast cancer
