# Supplementary material for: Prognostic impact of the glypican family of heparan sulfate proteoglycans on the survival of breast cancer patients
Source: J Cancer Res Clin Oncol. 2021 Mar 19;147(7):1937–55. doi: 10.1007/s00432-021-03597-4 (PMC8164625; doi:10.1007/s00432-021-03597-4)
Supplement: Supplementary file 1 — Supplementary file1 (DOCX 29 KB) [file 432_2021_3597_MOESM1_ESM.docx]

**Supplementary Material**

Table S1

| **Cell line** | **Tissue** | **Derived from** | **Cell type** | **Disease** | **Age (years)** | **Gender** | **Ethnicity** |
| --- | --- | --- | --- | --- | --- | --- | --- |
| **MCF-7** | Breast/ Mammary | metastatic site: pleural effusion | Epithelial | Adenocarcinoma | 69 | female | Caucasian |
| **T47D** | Breast/ Mammary | metastatic site: pleural effusion | Epithelial | Ductal Carcinoma | 54 | female | N/A |
| **SKBR3** | Breast/ Mammary | metastatic site: pleural effusion | Epithelial | Adenocarcinoma | 43 | female | Caucasian |
| **BT-474** | Breast/ Mammary | breast/duct | Epithelial | Ductal Carcinoma | 60 | female | Caucasian |
| **MDA-MB-231** | Breast/ Mammary | metastatic site: pleural effusion | Epithelial | Adenocarcinoma | 51 | female | Caucasian |
| **MDA-MB-453** | Breast/ Mammary | metastatic site: pericardial effusion | Epithelial | Metastatic Carcinoma | 48 | female | Caucasian |
| **MDA-MB-468** | Breast/ Mammary | metastatic site: pleural effusion | Epithelial | Adenocarcinoma | 51 | female | Black |

**Table S1. Major characteristics of cell lines.**

Table S2

| GPC1 | Forward: CATCGGGTGTGGAGAGTG  Reverse: TGAGCGTGTCCCTGTTGTC |
| --- | --- |
| GPC2 | Forward: CTGGGACACGACCTGGAC  Reverse: GCCATCCAGTCATCTGCATAC |
| GPC3 | Forward: CTGCTTCAGTCTGCAAGTATGG  Reverse: GTGGAGTCAGGCTTGGGTAG |
| GPC4 | Forward: AGTGTGGTCAGCGAACAGTG  Reverse: CAAACATATCATTCAGGGATTTCTC |
| GPC5 | Forward: GCCGCCCTGTAAGAACAC  Reverse: TCATTCCATGCTTCTCTTTGC |
| GPC6 | Forward: CCAGGCATAAGAAATTTGACG  Reverse: CATGTACAGCATGCCATAGGTC |

**Table S2. Sequences of Primer Pairs**

Table S3

| **Category** | **Description** | **Observed gene count** | **Strength** | **False discovery rate** | **Matching proteins in your network** |
| --- | --- | --- | --- | --- | --- |
| **Molecular function** | GO:0017134 - fibroblast growth factor binding | 4 | 2.16 | 0.00000472 | GPC1,FGFR4,FGFR1,FGFR2 |
|  | GO:0005007 - fibroblast growth factor-activated receptor activity | 3 | 2.65 | 0.00000902 | FGFR4,FGFR1,FGFR2 |
|  | GO:0005539 - glycosaminoglycan binding | 6 | 1.31 | 0.0000212 | FGF2,FGFR4,SHH,PTCH1,FGFR1,FGFR2 |
|  | GO:0016303 - 1-phosphatidylinositol-3-kinase activity | 4 | 1.84 | 0.0000212 | FGF2,FGFR4,FGFR1,FGFR2 |
|  | GO:0008201 - heparin binding | 5 | 1.37 | 0.0000609 | FGF2,FGFR4,PTCH1,FGFR1,FGFR2 |
|  | GO:0046934 - phosphatidylinositol-4,5-bisphosphate 3-kinase activity | 4 | 1.65 | 0.0000609 | FGF2,FGFR4,FGFR1,FGFR2 |
|  | GO:0043236 - laminin binding | 3 | 1.91 | 0.00014 | GPC1,NID1,SHH |
|  | GO:0005088 - Ras guanyl-nucleotide exchange factor activity | 5 | 1.19 | 0.00021 | FGF2,FGFR4,FGFR1,FGFR2,TRAPPC4 |
|  | GO:0097108 - hedgehog family protein binding | 2 | 2.7 | 0.00021 | HHIP,PTCH1 |
|  | GO:0090722 - receptor-receptor interaction | 2 | 2.58 | 0.00028 | FGF2,FGFR1 |
| **Cellular component** | GO:0043202 - lysosomal lumen | 10 | 1.9 | 8.46E-15 | GPC1,GPC2,SDC2,GPC4,SDC4,HSPG2,GPC6,GPC5,SDC1,GPC3 |
|  | GO:0005796 - Golgi lumen | 10 | 1.87 | 8.46E-15 | GPC1,GPC2,SDC2,GPC4,SDC4,HSPG2,GPC6,GPC5,SDC1,GPC3 |
|  | GO:0005775 - vacuolar lumen | 11 | 1.68 | 1.19E-14 | SDCBP,GPC1,GPC2,SDC2,GPC4,SDC4,HSPG2,GPC6,GPC5,SDC1,GPC3 |
|  | GO:0005576 - extracellular region | 20 | 0.78 | 5.93E-12 | SDCBP,GPC1,NID1,FGF2,GPC2,FGFR4,TDGF1,HHIP,SHH,GPC4,SDC4,HSPG2,GPC6,GPC5,GPC3,AFP,NOTUM,FGFR1,AHSG,FGFR2 |
|  | GO:0031012 - extracellular matrix | 10 | 1.42 | 6.19E-11 | GPC1,NID1,GPC2,SHH,GPC4,HSPG2,GPC6,GPC5,GPC3,AHSG |
|  | GO:0046658 - anchored component of plasma membrane | 6 | 1.99 | 1.32E-09 | GPC1,GPC2,GPC4,GPC6,GPC5,GPC3 |
|  | GO:0005794 - Golgi apparatus | 15 | 0.88 | 1.32E-09 | GPC1,GPC2,FGFR4,SDC2,GPC4,SDC4,HSPG2,GPC6,GPC5,SDC1,GOLM1,GPC3,AHSG,FGFR2,TRAPPC4 |
|  | GO:0005764 - lysosome | 11 | 1.15 | 1.71E-09 | SDCBP,GPC1,GPC2,SDC2,GPC4,SDC4,HSPG2,GPC6,GPC5,SDC1,GPC3 |
|  | GO:0062023 - collagen-containing extracellular matrix | 7 | 1.56 | 1.11E-08 | NID1,GPC2,GPC4,HSPG2,GPC6,GPC5,GPC3 |
|  | GO:0031225 - anchored component of membrane | 7 | 1.53 | 1.75E-08 | GPC1,GPC2,TDGF1,GPC4,GPC6,GPC5,GPC3 |
| **Biological processes** | GO:0006027 - glycosaminoglycan catabolic process | 11 | 2.13 | 1.01E-17 | GPC1,FGF2,GPC2,SDC2,GPC4,SDC4,HSPG2,GPC6,GPC5,SDC1,GPC3 |
|  | GO:0001523 - retinoid metabolic process | 10 | 1.94 | 1.43E-14 | GPC1,GPC2,SDC2,GPC4,SDC4,HSPG2,GPC6,GPC5,SDC1,GPC3 |
|  | GO:0006024 - glycosaminoglycan biosynthetic process | 10 | 1.87 | 2.9E-14 | GPC1,GPC2,SDC2,GPC4,SDC4,HSPG2,GPC6,GPC5,SDC1,GPC3 |
|  | GO:0006629 - lipid metabolic process | 16 | 1 | 1.53E-11 | GPC1,FGF2,GPC2,FGFR4,SHH,SDC2,GPC4,SDC4,HSPG2,GPC6,GPC5,SDC1,GPC3,AFP,FGFR1,FGFR2 |
|  | GO:0001657 - ureteric bud development | 8 | 1.85 | 3.65E-11 | FGF2,SHH,PTCH1,SDC4,SDC1,GPC3,FGFR1,FGFR2 |
|  | GO:0001822 - kidney development | 10 | 1.48 | 6.03E-11 | NID1,FGF2,SHH,PTCH1,SDC4,SDC1,GPC3,GLI2,FGFR1,FGFR2 |
|  | GO:0001763 - morphogenesis of a branching structure | 9 | 1.6 | 8.22E-11 | FGF2,TDGF1,HHIP,SHH,PTCH1,GPC3,GLI2,FGFR1,FGFR2 |
|  | GO:0044255 - cellular lipid metabolic process | 14 | 1.05 | 1.83E-10 | GPC1,FGF2,GPC2,FGFR4,SDC2,GPC4,SDC4,HSPG2,GPC6,GPC5,SDC1,GPC3,FGFR1,FGFR2 |
|  | GO:0061138 - morphogenesis of a branching epithelium | 8 | 1.58 | 2.31E-09 | FGF2,HHIP,SHH,PTCH1,GPC3,GLI2,FGFR1,FGFR2 |
|  | GO:0035295 - tube development | 12 | 1.06 | 7.94E-09 | FGF2,TDGF1,HHIP,SHH,PTCH1,SDC4,HSPG2,SDC1,GPC3,GLI2,FGFR1,FGFR2 |

**Table S3. Gene Ontology (GO) Enrichment Analysis associated with GPC1, GPC2, GPC3, GPC4, GPC5, GPC6.** The program STRING was used for this analysis.

Table S4

| **Genes** | **LN Status** | **Relapse Free Survival** | | |
| --- | --- | --- | --- | --- |
|  |  | **Number of cases** | **HR 95% CI** | **P value** |
| **GPC1** | positive | 1133 | 1.13 (0.93-1.38) | 0.22 |
|  | negative | 2020 | 0.91 (0.77-1.08) | 0.29 |
| **GPC2** | positive | 724 | 1.06 (0.83-1.37) | 0.6261 |
|  | negative | 496 | 1.07 (0.72-1.58) | 0.75 |
| **GPC3** | positive | 1133 | 0.91 (0.75-1.11) | 0.34 |
|  | negative | 2020 | 0.94 (0.79-1.11) | 0.45 |
| **GPC4** | positive | 1133 | 0.89 (0.73-1.09) | 0.25 |
|  | negative | 2020 | 0.87 (0.74-1.03) | 0.11 |
| **GPC5** | positive | 1133 | 1.03 (0.84-1.25) | 0.79 |
|  | negative | 2020 | 0.9 (0.76-1.07) | 0.23 |
| **GPC6** | positive | 724 | 1.02 (0.79-1.3) | 0.91 |
|  | negative | 496 | 1.23 (0.83-1.8) | 0.3 |

**Table S4.** Prognostic significance of the expression of glypicans on the relapse free survival of breast cancer patients stratified by lymph node (LN) status. HR = Hazard Ratio, CI = Confidence Interval.

Table S5

| **Identifier** | **Protein Name** |
| --- | --- |
| [A6NFA1](http://matrixdb.univ-lyon1.fr/cgi-bin/current/newPort?type=biomolecule&value=A6NFA1) | Metalloprotease TIKI2 |
| [CHEBI:28304](http://matrixdb.univ-lyon1.fr/cgi-bin/current/newPort?type=biomolecule&value=GAG_1) | Heparin |
| [O00560](http://matrixdb.univ-lyon1.fr/cgi-bin/current/newPort?type=biomolecule&value=O00560) | Syntenin-1 |
| [O00626](http://matrixdb.univ-lyon1.fr/cgi-bin/current/newPort?type=biomolecule&value=O00626) | C-C motif chemokine 22 |
| [O14625](http://matrixdb.univ-lyon1.fr/cgi-bin/current/newPort?type=biomolecule&value=O14625) | C-X-C motif chemokine 11 |
| [O14936](http://matrixdb.univ-lyon1.fr/cgi-bin/current/newPort?type=biomolecule&value=O14936) | Peripheral plasma membrane protein CASK |
| [O35182](http://matrixdb.univ-lyon1.fr/cgi-bin/current/newPort?type=biomolecule&value=O35182) | Mothers against decapentaplegic homolog 6 |
| [O43541](http://matrixdb.univ-lyon1.fr/cgi-bin/current/newPort?type=biomolecule&value=O43541) | Mothers against decapentaplegic homolog 6 |
| [O75063](http://matrixdb.univ-lyon1.fr/cgi-bin/current/newPort?type=biomolecule&value=O75063) | Glycosaminoglycan xylosylkinase |
| [O75077](http://matrixdb.univ-lyon1.fr/cgi-bin/current/newPort?type=biomolecule&value=O75077) | Disintegrin and metalloproteinase domain-containing protein 23 |
| [O75084](http://matrixdb.univ-lyon1.fr/cgi-bin/current/newPort?type=biomolecule&value=O75084) | Frizzled-7 |
| [O75197](http://matrixdb.univ-lyon1.fr/cgi-bin/current/newPort?type=biomolecule&value=O75197) | Low-density lipoprotein receptor-related protein 5 |
| [O75487](http://matrixdb.univ-lyon1.fr/cgi-bin/current/newPort?type=biomolecule&value=O75487) | Glypican-4 |
| [O75581](http://matrixdb.univ-lyon1.fr/cgi-bin/current/newPort?type=biomolecule&value=O75581) | Low-density lipoprotein receptor-related protein 6 |
| [O88569](http://matrixdb.univ-lyon1.fr/cgi-bin/current/newPort?type=biomolecule&value=O88569) | Heterogeneous nuclear ribonucleoproteins A2/B1 |
| [O94907](http://matrixdb.univ-lyon1.fr/cgi-bin/current/newPort?type=biomolecule&value=O94907) | Dickkopf-related protein 1 |
| [O94985](http://matrixdb.univ-lyon1.fr/cgi-bin/current/newPort?type=biomolecule&value=O94985) | Calsyntenin-1 |
| [O95166](http://matrixdb.univ-lyon1.fr/cgi-bin/current/newPort?type=biomolecule&value=O95166) | Gamma-aminobutyric acid receptor-associated protein |
| [O95716](http://matrixdb.univ-lyon1.fr/cgi-bin/current/newPort?type=biomolecule&value=O95716) | Ras-related protein Rab-3D |
| [O95980](http://matrixdb.univ-lyon1.fr/cgi-bin/current/newPort?type=biomolecule&value=O95980) | Reversion-inducing cysteine-rich protein with Kazal motifs |
| [P0CG48](http://matrixdb.univ-lyon1.fr/cgi-bin/current/newPort?type=biomolecule&value=P0CG48) | Polyubiquitin-C |
| [P00813](http://matrixdb.univ-lyon1.fr/cgi-bin/current/newPort?type=biomolecule&value=P00813) | Adenosine deaminase |
| [P01275](http://matrixdb.univ-lyon1.fr/cgi-bin/current/newPort?type=biomolecule&value=P01275) | Glucagon |
| [P01282](http://matrixdb.univ-lyon1.fr/cgi-bin/current/newPort?type=biomolecule&value=P01282) | VIP peptides |
| [P01286](http://matrixdb.univ-lyon1.fr/cgi-bin/current/newPort?type=biomolecule&value=P01286) | Somatoliberin |
| [P01303](http://matrixdb.univ-lyon1.fr/cgi-bin/current/newPort?type=biomolecule&value=P01303) | Pro-neuropeptide Y |
| [P02649](http://matrixdb.univ-lyon1.fr/cgi-bin/current/newPort?type=biomolecule&value=P02649) | Apolipoprotein E |
| [P02745](http://matrixdb.univ-lyon1.fr/cgi-bin/current/newPort?type=biomolecule&value=P02745) | Complement C1q subcomponent subunit A |
| [P02751](http://matrixdb.univ-lyon1.fr/cgi-bin/current/newPort?type=biomolecule&value=P02751) | Fibronectin |
| [P02778](http://matrixdb.univ-lyon1.fr/cgi-bin/current/newPort?type=biomolecule&value=P02778) | C-X-C motif chemokine 10 |
| [P04156](http://matrixdb.univ-lyon1.fr/cgi-bin/current/newPort?type=biomolecule&value=P04156) | Major prion protein |
| [P05067](http://matrixdb.univ-lyon1.fr/cgi-bin/current/newPort?type=biomolecule&value=P05067) | Amyloid-beta A4 protein |
| [P06748](http://matrixdb.univ-lyon1.fr/cgi-bin/current/newPort?type=biomolecule&value=P06748) | Nucleophosmin |
| [P07498](http://matrixdb.univ-lyon1.fr/cgi-bin/current/newPort?type=biomolecule&value=P07498) | Kappa-casein |
| [P08833](http://matrixdb.univ-lyon1.fr/cgi-bin/current/newPort?type=biomolecule&value=P08833) | Insulin-like growth factor-binding protein 1 |
| [P09543](http://matrixdb.univ-lyon1.fr/cgi-bin/current/newPort?type=biomolecule&value=P09543) | 2',3'-cyclic-nucleotide 3'-phosphodiesterase |
| [P09681](http://matrixdb.univ-lyon1.fr/cgi-bin/current/newPort?type=biomolecule&value=P09681) | Gastric inhibitory polypeptide |
| [P10082](http://matrixdb.univ-lyon1.fr/cgi-bin/current/newPort?type=biomolecule&value=P10082) | Peptide YY |
| [P10636](http://matrixdb.univ-lyon1.fr/cgi-bin/current/newPort?type=biomolecule&value=P10636) | Microtubule-associated protein Tau |
| [P10909](http://matrixdb.univ-lyon1.fr/cgi-bin/current/newPort?type=biomolecule&value=P10909) | Clusterin |
| [P11021](http://matrixdb.univ-lyon1.fr/cgi-bin/current/newPort?type=biomolecule&value=P11021) | Endoplasmic reticulum chaperone BiP |
| [P13385](http://matrixdb.univ-lyon1.fr/cgi-bin/current/newPort?type=biomolecule&value=P13385) | Teratocarcinoma-derived growth factor 1 |
| [P13501](http://matrixdb.univ-lyon1.fr/cgi-bin/current/newPort?type=biomolecule&value=P13501) | C-C motif chemokine 5 |
| [P14672](http://matrixdb.univ-lyon1.fr/cgi-bin/current/newPort?type=biomolecule&value=P14672) | Solute carrier family 2, facilitated glucose transporter member 4 |
| [P16220](http://matrixdb.univ-lyon1.fr/cgi-bin/current/newPort?type=biomolecule&value=P16220) | Cyclic AMP-responsive element-binding protein 1 |
| [P16284](http://matrixdb.univ-lyon1.fr/cgi-bin/current/newPort?type=biomolecule&value=P16284) | Platelet endothelial cell adhesion molecule |
| [P16860](http://matrixdb.univ-lyon1.fr/cgi-bin/current/newPort?type=biomolecule&value=P16860) | Natriuretic peptides B |
| [P18065](http://matrixdb.univ-lyon1.fr/cgi-bin/current/newPort?type=biomolecule&value=P18065) | Insulin-like growth factor-binding protein 2 |
| [P18509](http://matrixdb.univ-lyon1.fr/cgi-bin/current/newPort?type=biomolecule&value=P18509) | Pituitary adenylate cyclase-activating polypeptide |
| [P19784](http://matrixdb.univ-lyon1.fr/cgi-bin/current/newPort?type=biomolecule&value=P19784) | Casein kinase II subunit alpha' |
| [P19875](http://matrixdb.univ-lyon1.fr/cgi-bin/current/newPort?type=biomolecule&value=P19875) | C-X-C motif chemokine 2 |
| [P20366](http://matrixdb.univ-lyon1.fr/cgi-bin/current/newPort?type=biomolecule&value=P20366) | Protachykinin-1 |
| [P22626](http://matrixdb.univ-lyon1.fr/cgi-bin/current/newPort?type=biomolecule&value=P22626) | Heterogeneous nuclear ribonucleoproteins A2/B1 |
| [P22692](http://matrixdb.univ-lyon1.fr/cgi-bin/current/newPort?type=biomolecule&value=P22692) | Insulin-like growth factor-binding protein 4 |
| [P24592](http://matrixdb.univ-lyon1.fr/cgi-bin/current/newPort?type=biomolecule&value=P24592) | Insulin-like growth factor-binding protein 6 |
| [P24863](http://matrixdb.univ-lyon1.fr/cgi-bin/current/newPort?type=biomolecule&value=P24863) | Cyclin-C |
| [P27487](http://matrixdb.univ-lyon1.fr/cgi-bin/current/newPort?type=biomolecule&value=P27487) | Dipeptidyl peptidase 4 |
| [P30153](http://matrixdb.univ-lyon1.fr/cgi-bin/current/newPort?type=biomolecule&value=P30153) | Serine/threonine-protein phosphatase 2A 65 kDa regulatory subunit A alpha isoform |
| [P35052](http://matrixdb.univ-lyon1.fr/cgi-bin/current/newPort?type=biomolecule&value=P35052) | Glypican-1 |
| [P35998](http://matrixdb.univ-lyon1.fr/cgi-bin/current/newPort?type=biomolecule&value=P35998) | 26S Proteasome regulatory subunit 7 |
| [P41221](http://matrixdb.univ-lyon1.fr/cgi-bin/current/newPort?type=biomolecule&value=P41221) | Protein Wnt-5a |
| [P41743](http://matrixdb.univ-lyon1.fr/cgi-bin/current/newPort?type=biomolecule&value=P41743) | Protein kinase C iota type |
| [P42858](http://matrixdb.univ-lyon1.fr/cgi-bin/current/newPort?type=biomolecule&value=P42858) | Huntingtin |
| [P46379](http://matrixdb.univ-lyon1.fr/cgi-bin/current/newPort?type=biomolecule&value=P46379) | Large proline-rich protein BAG6 |
| [P46940](http://matrixdb.univ-lyon1.fr/cgi-bin/current/newPort?type=biomolecule&value=P46940) | Ras GTPase-activating-like protein IQGAP1 |
| [P48061](http://matrixdb.univ-lyon1.fr/cgi-bin/current/newPort?type=biomolecule&value=P48061) | Stromal cell-derived factor 1 |
| [P51654](http://matrixdb.univ-lyon1.fr/cgi-bin/current/newPort?type=biomolecule&value=P51654) | Glypican-3 |
| [P51671](http://matrixdb.univ-lyon1.fr/cgi-bin/current/newPort?type=biomolecule&value=P51671) | Eotaxin |
| [P55060](http://matrixdb.univ-lyon1.fr/cgi-bin/current/newPort?type=biomolecule&value=P55060) | Exportin-2 |
| [P56658](http://matrixdb.univ-lyon1.fr/cgi-bin/current/newPort?type=biomolecule&value=P56658) | Adenosine deaminase |
| [P56703](http://matrixdb.univ-lyon1.fr/cgi-bin/current/newPort?type=biomolecule&value=P56703) | Proto-oncogene Wnt-3 |
| [P56704](http://matrixdb.univ-lyon1.fr/cgi-bin/current/newPort?type=biomolecule&value=P56704) | Protein Wnt-3a |
| [P56706](http://matrixdb.univ-lyon1.fr/cgi-bin/current/newPort?type=biomolecule&value=P56706) | Protein Wnt-7b |
| [P57078](http://matrixdb.univ-lyon1.fr/cgi-bin/current/newPort?type=biomolecule&value=P57078) | Receptor-interacting serine/threonine-protein kinase 4 |
| [P57729](http://matrixdb.univ-lyon1.fr/cgi-bin/current/newPort?type=biomolecule&value=P57729) | Ras-related protein Rab-38 |
| [P60520](http://matrixdb.univ-lyon1.fr/cgi-bin/current/newPort?type=biomolecule&value=P60520) | Gamma-aminobutyric acid receptor-associated protein-like 2 |
| [P83916](http://matrixdb.univ-lyon1.fr/cgi-bin/current/newPort?type=biomolecule&value=P83916) | Chromobox protein homolog 1 |
| [P83917](http://matrixdb.univ-lyon1.fr/cgi-bin/current/newPort?type=biomolecule&value=P83917) | Chromobox protein homolog 1 |
| [P98086](http://matrixdb.univ-lyon1.fr/cgi-bin/current/newPort?type=biomolecule&value=P98086) | Complement C1q subcomponent subunit A |
| [P05067-PRO_0000000092](http://matrixdb.univ-lyon1.fr/cgi-bin/current/newPort?type=biomolecule&value=PFRAG_12_human) | Beta-amyloid peptide 1-42 |
| [P05067-PRO_0000000093](http://matrixdb.univ-lyon1.fr/cgi-bin/current/newPort?type=biomolecule&value=PFRAG_13_human) | Beta-amyloid peptide 1-40 |
| [Q5S007](http://matrixdb.univ-lyon1.fr/cgi-bin/current/newPort?type=biomolecule&value=Q5S007) | Leucine-rich repeat serine/threonine-protein kinase 2 |
| [Q5T9L3](http://matrixdb.univ-lyon1.fr/cgi-bin/current/newPort?type=biomolecule&value=Q5T9L3) | Protein wntless homolog |
| [Q6FHJ7](http://matrixdb.univ-lyon1.fr/cgi-bin/current/newPort?type=biomolecule&value=Q6FHJ7) | Secreted frizzled-related protein 4 |
| [Q6IQ23](http://matrixdb.univ-lyon1.fr/cgi-bin/current/newPort?type=biomolecule&value=Q6IQ23) | Pleckstrin homology domain-containing family A member 7 |
| [Q6K0P9](http://matrixdb.univ-lyon1.fr/cgi-bin/current/newPort?type=biomolecule&value=Q6K0P9) | Pyrin and HIN domain-containing protein 1 |
| [Q6P988](http://matrixdb.univ-lyon1.fr/cgi-bin/current/newPort?type=biomolecule&value=Q6P988) | Palmitoleoyl-protein carboxylesterase NOTUM |
| [Q6RFH8](http://matrixdb.univ-lyon1.fr/cgi-bin/current/newPort?type=biomolecule&value=Q6RFH8) | Double homeobox protein 4C |
| [Q6UXH1](http://matrixdb.univ-lyon1.fr/cgi-bin/current/newPort?type=biomolecule&value=Q6UXH1) | Cysteine-rich with EGF-like domain protein 2 |
| [Q8IVT5](http://matrixdb.univ-lyon1.fr/cgi-bin/current/newPort?type=biomolecule&value=Q8IVT5) | Kinase suppressor of Ras 1 |
| [Q8J025](http://matrixdb.univ-lyon1.fr/cgi-bin/current/newPort?type=biomolecule&value=Q8J025) | Protein APCDD1 |
| [Q8N0Z3](http://matrixdb.univ-lyon1.fr/cgi-bin/current/newPort?type=biomolecule&value=Q8N0Z3) | Spindle and centriole-associated protein 1 |
| [Q8ND30](http://matrixdb.univ-lyon1.fr/cgi-bin/current/newPort?type=biomolecule&value=Q8ND30) | Liprin-beta-2 |
| [Q9BSJ2](http://matrixdb.univ-lyon1.fr/cgi-bin/current/newPort?type=biomolecule&value=Q9BSJ2) | Gamma-tubulin complex component 2 |
| [Q9H0R8](http://matrixdb.univ-lyon1.fr/cgi-bin/current/newPort?type=biomolecule&value=Q9H0R8) | Gamma-aminobutyric acid receptor-associated protein-like 1 |
| [Q9H237](http://matrixdb.univ-lyon1.fr/cgi-bin/current/newPort?type=biomolecule&value=Q9H237) | Protein-serine O-palmitoleoyltransferase porcupine |
| [Q9H461](http://matrixdb.univ-lyon1.fr/cgi-bin/current/newPort?type=biomolecule&value=Q9H461) | Frizzled-8 |
| [Q9HD26](http://matrixdb.univ-lyon1.fr/cgi-bin/current/newPort?type=biomolecule&value=Q9HD26) | Golgi-associated PDZ and coiled-coil motif-containing protein |
| [Q9P121](http://matrixdb.univ-lyon1.fr/cgi-bin/current/newPort?type=biomolecule&value=Q9P121) | Neurotrimin |
| [Q9UEF7](http://matrixdb.univ-lyon1.fr/cgi-bin/current/newPort?type=biomolecule&value=Q9UEF7) | Klotho |
| [Q9UKG1](http://matrixdb.univ-lyon1.fr/cgi-bin/current/newPort?type=biomolecule&value=Q9UKG1) | DCC-interacting protein 13-alpha |
| [Q9ULT6](http://matrixdb.univ-lyon1.fr/cgi-bin/current/newPort?type=biomolecule&value=Q9ULT6) | E3 ubiquitin-protein ligase znrf3 |
| [Q9ULV1](http://matrixdb.univ-lyon1.fr/cgi-bin/current/newPort?type=biomolecule&value=Q9ULV1) | Frizzled-4 |
| [Q9ULW2](http://matrixdb.univ-lyon1.fr/cgi-bin/current/newPort?type=biomolecule&value=Q9ULW2) | Frizzled-10 |
| [Q9UP38](http://matrixdb.univ-lyon1.fr/cgi-bin/current/newPort?type=biomolecule&value=Q9UP38) | Frizzled-1 |
| [Q9Y5W5](http://matrixdb.univ-lyon1.fr/cgi-bin/current/newPort?type=biomolecule&value=Q9Y5W5) | Wnt inhibitory factor 1 |
| [Q9Y625](http://matrixdb.univ-lyon1.fr/cgi-bin/current/newPort?type=biomolecule&value=Q9Y625) | Glypican-6 |
| [Q9Z0J1](http://matrixdb.univ-lyon1.fr/cgi-bin/current/newPort?type=biomolecule&value=Q9Z0J1) | Reversion-inducing cysteine-rich protein with Kazal motifs |
| [Q9Z1B5](http://matrixdb.univ-lyon1.fr/cgi-bin/current/newPort?type=biomolecule&value=Q9Z1B5) | Mitotic spindle assembly checkpoint protein MAD2A |
| [Q53F19](http://matrixdb.univ-lyon1.fr/cgi-bin/current/newPort?type=biomolecule&value=Q53F19) | Nuclear cap-binding protein subunit 3 |
| [Q96GS6](http://matrixdb.univ-lyon1.fr/cgi-bin/current/newPort?type=biomolecule&value=Q96GS6) | Alpha/beta hydrolase domain-containing protein 17A |
| [Q00604](http://matrixdb.univ-lyon1.fr/cgi-bin/current/newPort?type=biomolecule&value=Q00604) | Norrin |
| [Q969D9](http://matrixdb.univ-lyon1.fr/cgi-bin/current/newPort?type=biomolecule&value=Q969D9) | Thymic stromal lymphopoietin |
| [Q01973](http://matrixdb.univ-lyon1.fr/cgi-bin/current/newPort?type=biomolecule&value=Q01973) | Inactive tyrosine-protein kinase transmembrane receptor ROR1 |
| [Q07325](http://matrixdb.univ-lyon1.fr/cgi-bin/current/newPort?type=biomolecule&value=Q07325) | C-X-C motif chemokine 9 |
| [Q12841](http://matrixdb.univ-lyon1.fr/cgi-bin/current/newPort?type=biomolecule&value=Q12841) | Follistatin-related protein 1 |
| [Q12959](http://matrixdb.univ-lyon1.fr/cgi-bin/current/newPort?type=biomolecule&value=Q12959) | Disks large homolog 1 |
| [Q13257](http://matrixdb.univ-lyon1.fr/cgi-bin/current/newPort?type=biomolecule&value=Q13257) | Mitotic spindle assembly checkpoint protein MAD2A |
| [Q13442](http://matrixdb.univ-lyon1.fr/cgi-bin/current/newPort?type=biomolecule&value=Q13442) | 28 kDa heat- and acid-stable phosphoprotein |
| [Q13467](http://matrixdb.univ-lyon1.fr/cgi-bin/current/newPort?type=biomolecule&value=Q13467) | Frizzled-5 |
| [Q13469](http://matrixdb.univ-lyon1.fr/cgi-bin/current/newPort?type=biomolecule&value=Q13469) | Nuclear factor of activated T-cells, cytoplasmic 2 |
| [Q13501](http://matrixdb.univ-lyon1.fr/cgi-bin/current/newPort?type=biomolecule&value=Q13501) | Sequestosome-1 |
| [Q13618](http://matrixdb.univ-lyon1.fr/cgi-bin/current/newPort?type=biomolecule&value=Q13618) | Cullin-3 |
| [Q13637](http://matrixdb.univ-lyon1.fr/cgi-bin/current/newPort?type=biomolecule&value=Q13637) | Ras-related protein Rab-32 |
| [Q14515](http://matrixdb.univ-lyon1.fr/cgi-bin/current/newPort?type=biomolecule&value=Q14515) | SPARC-like protein 1 |
| [Q15008](http://matrixdb.univ-lyon1.fr/cgi-bin/current/newPort?type=biomolecule&value=Q15008) | 26S Proteasome non-ATPase regulatory subunit 6 |
| [Q15051](http://matrixdb.univ-lyon1.fr/cgi-bin/current/newPort?type=biomolecule&value=Q15051) | IQ calmodulin-binding motif-containing protein 1 |
| [Q15369](http://matrixdb.univ-lyon1.fr/cgi-bin/current/newPort?type=biomolecule&value=Q15369) | Elongin-C |
| [Q16827](http://matrixdb.univ-lyon1.fr/cgi-bin/current/newPort?type=biomolecule&value=Q16827) | Receptor-type tyrosine-protein phosphatase O |
| [Q61091](http://matrixdb.univ-lyon1.fr/cgi-bin/current/newPort?type=biomolecule&value=Q61091) | Frizzled-8 |
| [Q62074](http://matrixdb.univ-lyon1.fr/cgi-bin/current/newPort?type=biomolecule&value=Q62074) | Protein kinase C iota type |
| [Q92913](http://matrixdb.univ-lyon1.fr/cgi-bin/current/newPort?type=biomolecule&value=Q92913) | Fibroblast growth factor 13 |
| [Q92973](http://matrixdb.univ-lyon1.fr/cgi-bin/current/newPort?type=biomolecule&value=Q92973) | Transportin-1 |
| [Q93097](http://matrixdb.univ-lyon1.fr/cgi-bin/current/newPort?type=biomolecule&value=Q93097) | Protein Wnt-2b |

**Table S5. Interactors with GPC1, GPC3, GPC4, GPC6 as determined by MatrixDB.** Combined network partners based on experiments and literature.
